# At baseline patients treated with esketamine have higher burden of disease than other patients with treatment resistant depression: Learnings from a population based study

**DOI:** 10.1002/da.23138

**Published:** 2021-01-21

**Authors:** M. Soledad Cepeda, David M. Kern, Carla M. Canuso

**Affiliations:** ^1^ Department of Epidemiology Janssen Research & Development Titusville New Jersey USA; ^2^ Department of Neuroscience Janssen Research & Development Titusville New Jersey USA

**Keywords:** burden of disease, esketamine, observational studies

## Abstract

**Introduction:**

It is critical to assess who is being treated with a new marketed drug like esketamine to understand how it is used in the real‐world setting and the effects of the medication.

**Methods:**

Retrospective analysis using two large U.S. health care databases that included commercially insured and Medicaid patients. Patients treated with esketamine were identified and their baseline characteristics described and compared with the baseline characteristics of patients with treatment resistant depression (TRD) and with patients undergoing transcranial magnetic stimulation (TMS). To quantify the differences, standardized mean differences were calculated.

**Results:**

In the commercially insured database, 418 patients were treated with esketamine and 830,047 patients were in the TRD group. Large differences in baseline characteristics were observed. Patients in the esketamine group were more likely to have severe depression, suicidal thoughts, and prior treatments with TMS or electroconvulsive therapy than the TRD control group. Patients in the esketamine group had more comorbid psychiatric conditions (anxiety disorder, posttraumatic stress disorders, substance use disorders) and higher exposure to antipsychotics, antiepileptics, hypnotics and sedatives. In terms of general health, patients in the esketamine group had many more outpatient visits, were more likely to have chronic pain and higher Charlson comorbidity scores, a predicator of mortality. Results were similar for both the Medicaid and TMS populations.

**Conclusion:**

Patients treated with esketamine have a higher burden of disease than other patients with TRD. In any real‐world comparative effectiveness or safety study these differences need to be understood and accounted for to produce valid results.

## 1 INTRODUCTION

Millions of individuals struggle from depression; in 2018 in the U.S. 17 million adults, or 7.2% of the adult population in the United States, had experienced a major depressive episode within the last year. Patients struggle with major depressive episodes often do not exhibit improvement with conventional antidepressant treatments, and 10% of patients newly diagnosed and treated for depression developed treatment resistant depression (TRD) within a year (Cepeda et al., 2018). Esketamine nasal spray was approved in March 2019 for the treatment of TRD in conjunction with an oral antidepressant and in August 2020 received additional approval to treat depressive symptoms in adults with major depressive disorder (MDD) with acute suicidal ideation or behavior. Esketamine is a nonselective *N*‐methyl d‐aspartate receptor antagonist that modulates glutamatergic transmission, (Daly et al., 2019). It was developed as an intranasal formulation.

The pattern of use of newly marketed medications, such as esketamine, in the real world will likely be different from what was observed in clinical trials. It has been shown that newly marketed drugs are commonly prescribed to patients whose clinical characteristics may have made them ineligible for the randomized clinical trials leading to the drugs ‘s approval (Munk et al., 2020). Patients in clinical trials are healthier and younger than real world patients (Leinonen et al., 2015; Mitchell et al., 2014; Schoenmaker & Van Gool, 2004) Therefore, once a new drug is on the market, it is critical to understand who is being treated to understand the real world effects of the drug.

An examination of who is being treated with esketamine in the real world will enhance the understanding of the efficacy and safety of esketamine and inform the design of any comparative effectiveness research. Claims health care databases are an ideal source to characterize the patients being administered esketamine. These databases contain information on medical conditions, procedures, and health care utilization over time. We sought to determine the baseline characteristics of patients currently being treated with esketamine and compare those baseline characteristics with the characteristics of patients with TRD who have not been treated with esketamine, subsequently referred to as the TRD control group.

## 2 MATERIALS AND METHODS

We conducted a retrospective analysis using two large US healthcare databases IBM MarketScan® Commercial Database (CCAE) and MarketScan® Multi‐State Medicaid Database (MDCD).

CCAE is a large U.S. claims database that includes data from at least 140 million individuals enrolled in employer‐sponsored insurance health plans. The data includes adjudicated health insurance claims as well as enrollment data from large employers and health plans who provide private healthcare coverage to employees, their spouses, and dependents. We used the latest version at our disposal that covered claims to March 2020.

MDCD is a large US database of adjudicated health insurance claims for more than 29 million Medicaid enrollees from multiple states. It includes hospital discharge diagnoses, outpatient diagnoses and procedures, and outpatient pharmacy claims. We used the latest version at our disposal that covered claims to December 2019.

In both databases, data elements are outpatient pharmacy dispensing claims (coded with National Drug Codes), inpatient and outpatient medical claims which provide procedure codes (coded in CPT‐4, HCPCs, ICD‐9‐CM, or ICD‐10‐PCS) and diagnosis codes (coded in ICD‐9‐CM or ICD‐10‐CM).

### 2.1 Esketamine and TRD identification

Two cohorts were created: patients treated with esketamine and patients with TRD who did not receive esketamine. Exposure to esketamine was identified through ingredient concept and G codes, see Table 1.

**Table 1:** Esketamine and TMS identification

Concept ID	Description
2119365 (Ingredient)	Esketamine
G2083 (Procedure)	Office or other outpatient visit for the evaluation and management of an established patient that requires the supervision of a physician or other qualified health care professional and provision of greater than 56 mg esketamine nasal self‐administration
G2082 (Procedure)	Office or other outpatient visit for the evaluation and management of an established patient that requires the supervision of a physician or other qualified health care professional and provision of up to 56 mg of esketamine nasal self‐administration
0310T (Procedure)	Motor function mapping using noninvasive navigated transcranial magnetic stimulation (nTMS) for therapeutic treatment planning, upper and lower extremity
0161T (Procedure)	Therapeutic repetitive transcranial magnetic stimulation treatment delivery and management, per session
0160T (Procedure)	Therapeutic repetitive transcranial magnetic stimulation treatment planning
90867 (Procedure)	Therapeutic repetitive transcranial magnetic stimulation (TMS) treatment; initial, including cortical mapping, motor threshold determination, delivery and management
90868 (Procedure)	Therapeutic repetitive transcranial magnetic stimulation (TMS) treatment; subsequent delivery and management, per session
90869 (Procedure)	Therapeutic repetitive transcranial magnetic stimulation (TMS) treatment; subsequent motor threshold redetermination with delivery and management

In clinical practice, patients with MDD who, despite having received treatment with at least two antidepressants given at adequate doses for an adequate duration in the current episode, have not responded are considered to have TRD. Implementing such a definition in claims databases is challenging because it is difficult to ascertain why medications were changed or stopped—improvement, lack of efficacy, or adverse events (Cepeda et al., 2018; Fife et al., 2018). Nonetheless, there is a validated definition of TRD which can be implemented in claims databases (Cepeda et al., 2018).

Using this definition, TRD was defined as present for patients with a diagnosis of MDD who were dispensed three distinct antidepressants or one antidepressant and one antipsychotic in 1 year. For example, a patient with depression who received sertraline, escitalopram, and amitriptyline within a year after the diagnosis of depression is considered to have TRD. Similarly, a patient with depression who received sertraline and quetiapine within a year after the diagnosis of depression is considered to have TRD. This definition of TRD proved to be superior to definitions that attempted to define TRD based on adequacy of treatment dose and duration (Cepeda et al., 2018). In the TRD control group we excluded patients with exposure to esketamine.

The index date was the date of the exposure to esketamine or when the patients were identified to have TRD (received the third antidepressant or the antipsychotic). We included everyone treated with esketamine in the database (we did not require that patients in the esketamine group have TRD).

### 2.2 Common data model (CDM)

The databases were converted to the Observational Medical Outcomes Partnership (OMOP) CDM (Stang et al., 2010). In the OMOP vocabulary drugs and conditions are referred to by concepts. The OMOP vocabulary provides relationships and ancestry relationships between concepts and extensive mapping to a variety of classification systems (Reich et al., 2012), so that drugs and conditions can be grouped at specific levels of a hierarchy in a specific classification system. A series of standardized analytic tools have been developed against the OMOP CDM as part of the Observational Health Data Sciences and Informatics collaborative (Hripcsak et al., 2015).

Medical conditions were defined using SNOMED (Systematized Nomenclature of Medicine‐Clinical Terms). SNOMED is a standardized, multilingual vocabulary of clinical terminology that is used by physicians and other health care providers for the electronic exchange of clinical health information (Reich et al., 2012). Medications were grouped using Anatomical Therapeutic Chemical Classification System.

### 2.3 Baseline characteristics

Since a comparison of medical comorbidities, medications, and procedures between esketamine and TRD control groups could include thousands of variables, we prioritized the reporting to characteristics that would suggest severity or refractoriness of the major depression and overall health care status. These variables included age, gender, psychiatric conditions, and to summarize the nonpsychiatric medical conditions, we calculated the Charlson comorbidity index. This index is a weighted sum of the presence of 19 medical conditions that affect the risk of mortality. Each condition is assigned a weight from 1 to 6, with higher weights indicating greater severity and higher risk of mortality (Charlson et al., 1987). In terms of medications, we included exposure to antiepileptics, anticonvulsants, hypnotics and sedatives. We also calculated as a proxy of refractoriness to treatment the proportion of patients who received at least four distinct antidepressants at two different time points (any time prior or in the previous year). As for nonpharmacological treatment procedures, we included transcranial magnetic stimulation (TMS) and electroconvulsive therapy (ECT). In addition, we included the total number of visits to the health care system in the year before. For the rest of the characteristics, we assessed them any time before the index date.

### 2.4 Statistics

To compare the baseline characteristics between the esketamine and the TRD control group, we calculated standardized mean differences (SMD). SMD is the difference in prevalence of a specific characteristic in the two cohorts divided by the *SD* of the difference. A large absolute value SMD on a covariate is an indication of a significant disparity in the proportion of patients with the covariate between the two groups. An SMD more than 0.1 has been used as an ad hoc heuristic for what constitutes “large” (Austin, 2009).

### 2.5 Post hoc analysis

In addition to comparing the characteristics of esketamine patients with the TRD control group, we added as a comparator patients undergoing TMS, a treatment that, similar to esketamine, is reserved for patients with more refractory disease. TMS devices received FDA clearance for adults with MDD who had not seen success with at least one antidepressant in 2008 (McClintock et al., 2018).

Patients who had undergone a TMS procedure recorded for the first time in the database were included in the TMS group, Table 1 has the codes used to identify TMS exposure. In addition, these patients had to have a diagnosis of major depression withing 1 year before TMS exposure. The index date was the day of the first TMS.

## 3 RESULTS

In CCAE, a total of 418 patients were treated with esketamine and 830,047 patients met the definition of having TRD and were not treated with esketamine in the CCAE database.

Patients in the esketamine group were slightly older and included a higher proportion of men (Table 2). All other baseline characteristics were substantially different in the esketamine group compared to the TRD control group (SMD >0.10) indicating a higher severity of illness and a higher burden of disease.

**Table 2:** Comparison of baseline characteristics of patients in the esketamine group, the treatment resistant depression (TRD) control group and the transcranial magnetic stimulation (TMS) group

	Esketamine	TRD	Standardized mean difference	TMS	Standardized mean difference
Total number of patients	418	830,047	–	7529	–
Age (mean ± *SD*)	43.53 ± 13.12	40.56 ± 14.62	0.15	43.44 ± 13.43	0.005
Women (%)	61.00	69.49	0.07	65.72	0.04
Men (%)	39.00	30.51	0.10	34.28	0.05
Severe depression (%) (any time)	79.19	29.28	0.48	93.80	−0.11
Generalized anxiety disorder (%) (any time)	71.53	26.55	0.45	59.25	0.11
Chronic pain (%) (any time)	39.0	11.50	0.39	24.17	0.19
Posttraumatic stress disorder (%) (any time)	26.56	6.65	0.34	18.86	0.11
Suicidal thoughts (%) (any time)	27.03	6.93	0.34	18.52	0.13
ADHD (%) (any time)	33.97	11.74	0.33	27.04	0.09
Obstructive sleep apnea syndrome (%) (any time)	29.19	9.41	0.32	24.92	0.06
Psychoactive substance use disorder (%) (any time)	20.81	8.88	0.22	14.30	0.11
Charlson index (mean ± *SD*) (any time)	1.55 ± 2.07	0.94 ± 1.88	0.21	1.30 ± 1.95	0.08
Antipsychotics (Other) (%) (any time)	65.07	26.56	0.40	48.23	0.16
Psychostimulants, agents used for ADHD (%) (any time)	59.09	22.73	0.40	48.81	0.10
Antiepileptics (%) (any time)	84.93	44.38	0.36	71.58	0.11
Hypnotics and sedatives (%) (any time)	58.61	37.98	0.21	50.18	0.08
At least four distinct antidepressants (%) (any time)	62.92	26.14	0.39	51.51	0.13
At least four distinct antidepressants (%) (year before)	12.92	7.50	0.12	10.96	0.04
Transcranial magnetic stimulation (%) (any time)	23.21	0.13	0.48	–	–
Electroconvulsive therapy (%) (any time)	11.24	0.22	0.34	5.30	0.15
Number of outpatient visits (mean ± *SD*) (year before)	50.83 ± 42.51	27.14 ± 22.74	0.49	42.63 ± 31.31	0.15

*Note:* Data source (CCAE).

Abbreviation: ADHD, attention deficit hyperactivity disorder.

The proportion of patients with a history of severe major depression was much higher in the esketamine group than in the TRD control group (79.19% and 29.28%, respectively). Patients in the esketamine group were more likely to have a history of suicidal thoughts, and to have received treatment with TMS and ECT than the TRD control group. Approximately 23% of patients in the esketamine group had undergone TMS compared to less than 1% in the TRD control group. Patients in the esketamine group also had a history of more psychiatric comorbidities such as anxiety disorder, posttraumatic stress disorder, and substance use disorder than the TRD control group. For example, approximately 71% of patients in the esketamine group had a diagnosis of generalized anxiety disorder compared to 26% in the TRD control group, Table 2.

The esketamine group also had a higher exposure to antipsychotics, antiepileptics, hypnotics, and sedatives at baseline. For example, in the esketamine group approximately 65% of patients had prior exposures to antipsychotics compared to 26% in the TRD control group. In terms of depression refractoriness, the percentage of patients with at least four distinct antidepressants (any time in the history or a year before index date) was higher in the esketamine group than in the TRD control group, Table 2.

In terms of general health, patients in the esketamine group had many more outpatient visits to health care providers than the TRD control group. The mean number of visits in the previous year was 50.83 versus 27.14 for the esketamine and TRD control groups respectively. In terms of nonpsychiatric conditions, patients in the esketamine group were more likely to have chronic pain, and higher Charlson comorbidity index score, Table 2.

In MDCD, a total of 50 patients were treated with esketamine and 312,459 patients met the definition of having TRD. Medicaid patients with TRD had a higher burden of disease than commercially insured patients with TRD. Patients in the Medicaid TRD control group had more chronic pain, posttraumatic stress disorder, suicidal thoughts, and substance use disorder than the commercially insured TRD control group. The Charlson comorbidity score was also higher and they had more visits to health care providers than commercially insured TRD patients, Table 3.

**Table 3:** Comparison of baseline characteristics of patients in the esketamine group, and the treatment resistant depression (TRD) group

	Esketamine	TRD	Standardized mean difference
Total number of patients	50	312459	
Age (mean ± *SD*)	40.68 ± 11.69	36.65 ± 15.94	0.20
Women (%)	50.0	72.07	0.20
Men (%)	50.0	27.3	0.25
Severe depression (%) (any time)	74.00	25.99	0.48
Generalized anxiety disorder (%) (any time)	74.00	27.18	0.46
Chronic pain (%) (any time)	58.00	28.46	0.31
Posttraumatic stress disorder (%) (any time)	32.00	17.94	0.20
Suicidal thoughts (%) (any time)	42.00	13.48	0.38
ADHD (%) (any time)	34.00	16.46	0.24
Obstructive sleep apnea syndrome (%) (any time)	38.00	9.65	0.41
Psychoactive substance use disorder (%) (any time)	38.00	15.82	0.30
Charlson index (mean ± *SD*) (any time)	1.68 ± 2.27	1.52 ± 2.47	0.05
Antipsychotics (Other) (%) (any time)	66.00	32.21	0.34
Psychostimulants, agents used for ADHD (%) (any time)	46.00	22.82	0.28
Antiepileptics (%) (any time)	86.00	51.09	0.30
Hypnotics and sedatives (%) (any time)	46.00	32.46	0.15
At least 4 distinct antidepressants (%) (any time)	62.0	20.42	0.45
At least 4 distinct antidepressants (%) (year before)	6	7.65	0.04
Transcranial magnetic stimulation (%) (any time)	2.00	0.02	0.14
Electroconvulsive therapy (%) (any time)	6.00	0.09	0.24
Number of outpatient visits (mean ± *SD*) (year before)	53.62 ± 46.87	46.37 ± 63.11	0.09

*Note:* Data source: Medicaid.

Abbreviation: ADHD, attention deficit hyperactivity disorder.

Similar to the esketamine findings in CCAE, in MDCD baseline characteristics were substantially different in the esketamine group compared to the TRD control group, patients in the esketamine group had a higher severity of illness and a higher burden of disease.

### 3.1 Pos hoc analysis

A total of 7529 patients were in the TMS group. TMS and esketamine patients were similar in age and sex. Patients in the TMS group had more severe depression than patients in the esketamine group (93.80% vs. 79.19%). Psychiatric comorbidities were more common in esketamine group than the TMS group as well as previous exposure to ECT (11.24% vs. 5.30%). The number of outpatient visits was also higher in the esketamine group than in the TMS group (53 visits in the previous year vs. 43), Table 2.

## 4 DISCUSSION

We compared the baseline characteristics of patients currently being treated with esketamine in real world settings to other patients with TRD and with subjects undergoing TMS. Esketamine treated patients were sicker, had a more refractory depression and had a much higher burden of disease than patients in the TRD control group. This is very important as it is well established that patients with TRD already have a severe burden of disease, are more symptomatic and exhibit worse outcomes (more suicides attempts and higher mortality) than patients with major depression (Benson et al., 2020; Feldman et al., 2013; Li et al., 2019).

In this study we captured medical conditions, procedures and medication use. However, information on race ethnicity, economic well‐being and social support were not reported as such data are often absent in claims databases. Such information could provide a more comprehensive picture of who is exposed to esketamine. To address this limitation, we included in addition to the commercial insured population, a Medicaid population. Overall, the TRD Medicaid patients had a higher burden of disease than the TRD commercially insured population. Other research concurs, it has been found that Medicaid patients had a higher risk of rehospitalization for suicidal ideation or suicidal attempts and had higher number of psychiatric and medical comorbidities than the commercially insured population (Cepeda et al., 2020; Kern et al., 2020). Despite these findings, esketamine treated patients, similar to the commercially insured population, had a higher burden of disease than the TRD control group, although there was a small number of patients treated with esketamine.

Esketamine treated patients and TMS patients share similarities in how they interact with the health care system. Esketamine and TMS often require prior authorizations and attestation of diagnosis and previous treatments. The comparison of baseline characteristics between esketamine treated patients and patients undergoing TMS portray similar results, esketamine patients had a higher burden disease, with one exception, the proportion of patients with severe depression. TMS authorization often requires that patients have severe major depression, which could explain the higher proportion of patients with diagnosed severe depression.

Our findings indicate that esketamine is being provided for a subgroup of patients with high burden of disease who are more severely ill than typical patients with TRD or patients undergoing TMS with the exception of severe depression which is higher in the TMS patients. These findings may reflect the level of commitment needed by patients and their providers to receive esketamine due to the required Risk Evaluation and Mitigation Strategy (REMS) program. The purpose of the REMS program is to mitigate potential risks of serious adverse outcomes associated with sedation, dissociation, and misuse/abuse of esketamine. Patients need to register, and pharmacies and healthcare settings that dispense esketamine must be certified. The REMS program includes restricted distribution and requires esketamine to only be dispensed and administered to patients in a medically supervised healthcare setting that monitors these patients for at least 2 h, and patients cannot drive until the next day after restful sleep. Additionally, current coverage of treatment and monitoring of esketamine nasal spray by insurers in the U.S. varies by plan and region, with many plans requiring prior authorization, a factor that may have limited access and led to use of esketamine later in the line of treatment after other treatment options have been used.

Patients on esketamine were more commonly exposed to antipsychotics and antiepileptics than patients in the TRD and TMS group. These medications are commonly used as antidepressant adjuncts (Kern et al., 2020; Vigo & Baldessarini, 2009) and reinforce the finding that patients on esketamine are more symptomatic than patients in the other comparator groups. Similarly, patients on esketamine had a history of being on hypnotics, sedatives and psychostimulants. The esketamine label warns about the concomitant use of central nervous system depressants as it may increase the risk of sedation during esketamine administration or the concomitant presence of psychostimulants as it may increase blood pressure during esketamine administration. The esketamine label also recommends monitoring for signs of abuse of patients at higher risk of abuse, 20% of esketamine treated patients had history of substance use disorder.

Since patients treated with esketamine are more severely ill and have a higher burden of diseases than other patients with TRD or patients with refractory depression such as the ones undergoing TMS, any comparative effectiveness research needs to address the substantial baseline differences, otherwise the outcomes assessed may reflect differences in the baseline characteristics of the patients treated instead of the effect of esketamine. Since the differences include a variety of variables: psychiatric and nonpsychiatric medical conditions, number of visits to health care providers, products and medications, any adjustment or control for confounding would require the use of techniques that would allow to control for many variables simultaneously such as propensity scores (Cepeda et al., 2003). Propensity scores also allow researchers to assess comparability of the groups as a whole, instead of just examining a few variables.

Since esketamine has only been on the market for a short period of time and a small number of patients have been treated, the profile of patients receiving esketamine may change overtime. Therefore similar analyses should be conducted as esketamine use increases in clinical practice.

## 5 CONCLUSION

It is critical to understand who is being treated with a newly marketed medication like esketamine nasal spray to understand its real world effects. Esketamine is currently being administered to patients with a high burden of disease that includes psychiatric and nonpsychiatric medical conditions. These findings are critical for the design and interpretation of future real‐world comparative effectiveness research as well as for safety evaluations, as baseline characteristics can influence these important outcomes in clinical practice.

## CONFLICT OF INTERESTS

All authors are employees of Janssen Research & Development, LLC and are stockholders of Johnson & Johnson, Inc.

## Data Availability

The source data for this study were licensed by Johnson & Johnson from IBM MarketScan® Commercial Database (CCAE), and hence we are not allowed to share the licensed data publicly. However, the same data used in this study are available for purchase by contracting the database owner (contact at: https://www.ibm.com/watson-health/products).
